# Effect of different brushing parameters on erosive tooth wear in primary bovine enamel and dentin

**DOI:** 10.1371/journal.pone.0302261

**Published:** 2024-04-16

**Authors:** Philipp Kanzow, Corinna Witt, Clemens Lechte, Sarah Barke, Bianca Rohland, Alexandra Schmidt, Annette Wiegand

**Affiliations:** Department of Preventive Dentistry, Periodontology and Cariology, University Medical Center Göttingen, Göttingen, Germany; Federal University of Minas Gerais: Universidade Federal de Minas Gerais, BRAZIL

## Abstract

This in-vitro study aimed to analyse the effect of brushing and different brushing parameters (kind of toothpaste, kind of toothbrush, brushing force) on erosive tooth wear of primary bovine enamel and dentin. Specimens were prepared from primary bovine enamel or dentin (each group n = 12) and cyclically eroded (6 × 60 s/d, citric acid, pH 2.4) and brushed with children’s toothbrushes (2 × 15 s/d) over 5 days. The brushing parameters under investigation were: toothpaste (fluoridated, fluoride-free), toothbrush (manual; rotating-oscillating and sonic, each at two different activation modes) and brushing force (1 N, 2 N). Specimens that were only eroded and not brushed served as controls. Enamel and dentin wear was quantified using widefield confocal microscopy. Statistical analysis was performed using three- and one-way ANOVAs followed by Scheffe’s (enamel) or Tamhane’s (dentin) post-hoc tests (p < 0.05). Brushing with the fluoridated toothpaste was able to significantly reduce erosive wear in enamel (by 15 to 37%, 6 of 10 groups) and in dentin (by 58 to 72%, all groups), while brushing with the fluoride-free toothpaste was not different from the controls. Considering the kind of toothpaste and brushing force, slight differences between the toothbrushes were observed in enamel, but not in dentin. Within the same toothbrush and activation mode, almost no differences between 1 and 2 N brushing force were detected. In conclusion, erosive tooth wear on primary bovine dental hard tissue mainly depends on the kind of toothpaste, rather than on the kind of toothbrush and the brushing force.

## Introduction

Erosive tooth wear is a prevalent condition in preschool children [1]. Previous studies have shown that primary dental hard tissues might be more prone to erosion [2] and erosive tooth wear than permanent enamel and dentin [3–5]. However, while the effect of toothbrushing on the development of erosive tooth wear has been intensively studied on permanent teeth [6], information on the interaction of toothbrushing and erosion on primary teeth is hardly available [3, 7].

Toothpastes are an ideal carrier for active substances, namely fluorides, which are able to reduce erosion by forming a CaF_2_-like layer on the tooth surface, which offers some temporary protection against acids. A recent study has shown that brushing with fluoride containing children’s toothpastes was able to prevent erosive tooth wear in primary dental hard tissue, especially in dentin, where even toothpastes with a lower fluoride concentration (500 ppm) were effective in reducing erosive wear [8]. However, from studies performed in permanent teeth it is known that brushing as such might reduce the protective effect of fluoride toothpastes compared to the toothpaste application as slurry, as particle type and size, wettability or calcium and phosphate concentration of the toothpastes partly counteract the effects of fluorides [9–11]. Moreover, the toothbrush itself, filament stiffness and the applied brushing force might also modify the abrasion of eroded dental hard tissue [12–15].

The interplay between erosion on the one hand, and the kind of toothpaste, the kind of toothbrush and the brushing force on the other hand has not been systematically analysed so far for primary dental hard tissue, although it was shown that primary dental hard tissue behaves differently from permanent enamel and dentin with regard to erosion and combined erosion/abrasion [5, 16].

As powered toothbrushes for children became increasingly popular and are frequently used by children (or parents) for cleaning children’s teeth [17, 18], this study aimed to analyse the effect of powered and manual toothbrushes for children applied at two different brushing forces and with a fluoride-containing or a fluoride-free children’s toothpaste on erosive tooth wear of primary enamel and dentin. The null hypothesis was that the kind of toothpaste (fluoridated vs. fluoride-free), the kind of toothbrush (manual vs. rotating-oscillating, sensitive mode vs. rotating-oscillating, daily clean mode vs. sonic, sensitive mode vs. sonic, power mode) and the brushing force (1 N vs. 2 N) has no impact on surface loss of eroded primary bovine enamel and dentin.

## Materials and methods

### Sample size calculation and preparation of specimens

As data regarding the clinically meaningful difference in surface loss are not available, sample size calculation was based on anticipated means derived from a preliminary study with the same design: Enamel loss by erosion amounted to 39.8 ± 2.2 μm and was reduced by brushing with a fluoridated children’s toothpaste to 35.7 ± 2.3 μm [8]. Considering that α = 0.0002381 (adjusted for multiple testing, 21 subgroups) and 1-β = 0.80, a group size of n = 12 was calculated (http://clincalc.com/stats/samplesize.aspx).

Specimens were prepared by a single operator (CW). Enamel and dentin specimens (each n = 252) were prepared from calves’ teeth that were taken from slaughterhouses as waste products in the slaughter process. Specimens were taken from the crown with water-cooled diamond-coated trepanning drills (inner diameter: 2.7 mm; custom-made, Gebr. Brasseler, Germany) and embedded in resin (Paladur; Kulzer, Germany). On average, 3 to 4 specimens per tooth were obtained. For enamel specimens, the surface was polished using water-cooled sandpaper (WS flex 18C grit 1.200; Hermes, Germany; silicon carbide grit 4.000; Buehler, Germany). To obtain dentin specimens, the enamel layer was completely removed and then polished as described above. No specimens were lost throughout the study, and specimens were randomly distributed among the experimental groups.

To allow for exact repositioning of the specimens during surface analyses by widefield confocal microscopy, holes were prepared into the acrylic resin surfaces. After baseline surface analysis, the outer thirds of the surfaces, including the holes, were covered with tape to protect the reference areas that are necessary for superimposition of widefield confocal microscopical images. These identification marks were covered by the tape throughout the experiment. After the experiment, the tape was removed for final surface analysis.

### Study design

**Fig 1:**
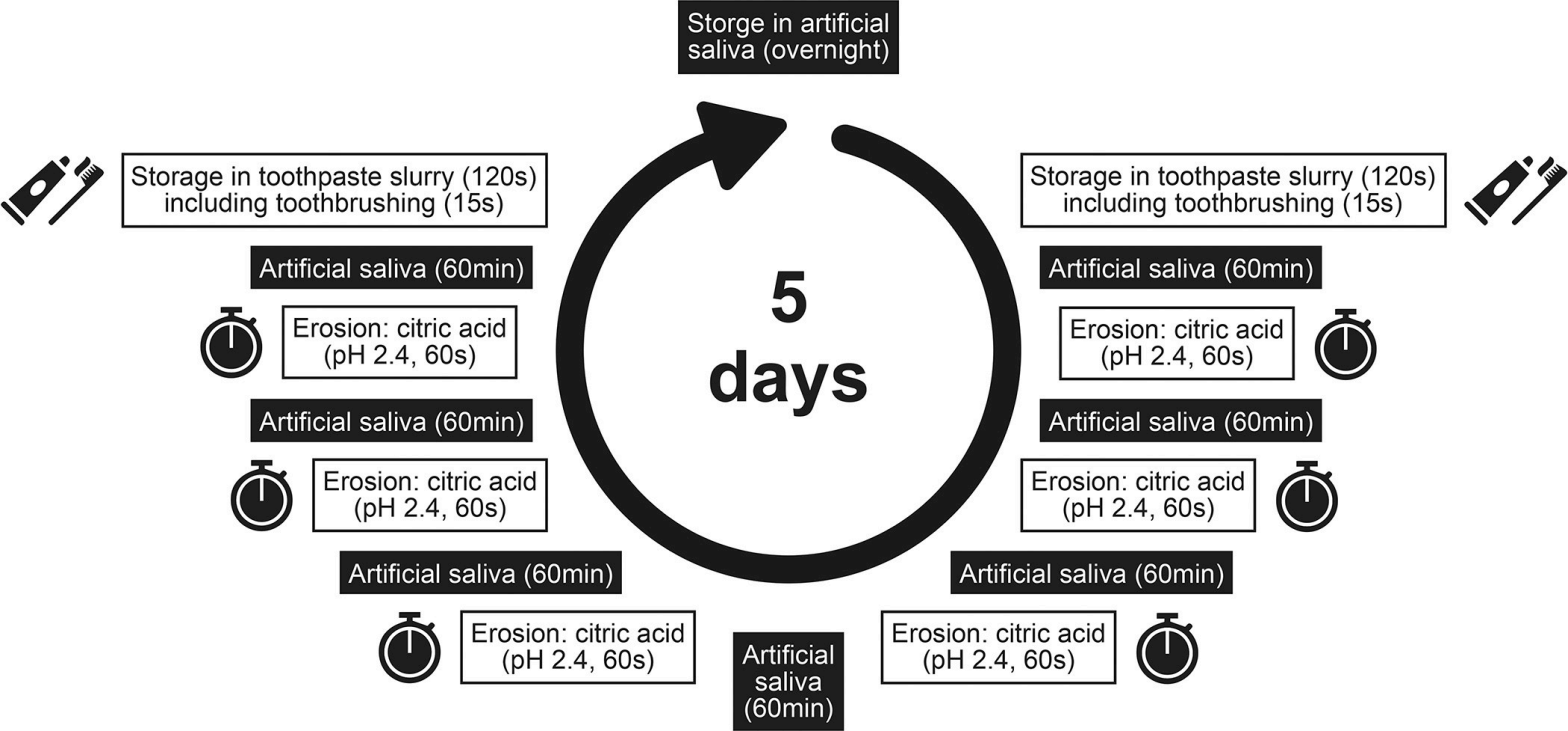
Study design.

The study design is depicted in Fig 1 and oriented on a previous study [8] and the recommendations for performing erosion-abrasion experiments [19].

Specimens were stored in distilled water prior to the 5-day erosive cycling experiment. Enamel and dentin specimens were subjected to erosion with citric acid (pH 2.4, 60 s, 1 ml) six times daily. Between the cycles (60 min) and overnight the specimens were stored in artificial saliva [20]. Erosion-only specimens served as control, while specimens in the experimental groups were subjected to toothbrushing: Daily, one hour before the first and one hour after the last erosion, the specimens were brushed in an automatic brushing machine (Willytec, Germany) for 15 s with children’s toothbrushes. The manual toothbrush (Dr. Best children toothbrush up to 7 years, GlaxoSmithKline Consumer Healthcare, Germany) was applied with linear reciprocating movements (80/min) [21, 22] at 1 or 2 N brushing force. The activated power toothbrushes were a rotating-oscillating (Oral-B Kids, Procter&Gamble, Germany; sensitive mode [7,100/min] or daily clean mode [7,600/min], age recommendation: 3 years and older) and a sonic toothbrush (Playbrush Smart Sonic, Playbrush, Austria; sensitive [16,000/min] or power mode [18,000/min], age recommendation: 3 years and older), that were applied activated with 20 linear back and forth movements/min [21, 22] at 1 and 2 N brushing force. Brushing was performed with a fluoridated (elmex Kinder, CP GABA, Germany; 1000 ppm fluoride, RDA: 70–80) or fluoride-free toothpaste (Weleda Kinderzahngel, Weleda, Germany; RDA: 60) for children. The toothpaste was applied as slurry that was prepared by mixing the respective toothpaste with water (1:2) [23]. Specimens were immersed in 3 ml of the toothpaste slurry for a total of 120 s. During this time period, specimens were brushed for 15 s.

### Surface analysis

Surface analysis was performed by two experienced operators (SB, BR) using widefield confocal microscopy (SmartProof 5, Zeiss, Germany) and ZEN Smartproof HF2 1.0 (Zeiss, Germany) for image acquisition. Of each enamel or dentin specimen, an 1125 × 4500 μm^2^ image with 10x magnification (C Epiplan-Apochromat 10×/0.4 objective lens) was taken before and after the experiment, and the surface profile was determined. Image acquisition was performed with a fast scan (150–200 z-levels) in low resolution in HDR mode. For image processing and evaluation, ConfoMap 7.4.8076 (Zeiss, Germany) was used. Further analyses of each image were performed, after the underground was leveled with a least-square method using rotation. The background noise was minimised by removing outliers. A three-fold determination of the surface loss of each specimen was done after superimposition of the surface profiles before and after the experiment. Surface loss was determined over a length of 0.5 mm in the middle of the specimen.

### Statistical analysis

Enamel and dentin loss (mean ± standard deviation) was calculated for all groups. Normal distribution of the data was tested using Kolmogorov-Smirnov and Shapiro-Wilk tests. As most groups presented a normal distribution, three-way ANOVAs were performed for enamel and dentin with the kind of toothpaste (fluoridated vs. fluoride-free), the kind of toothbrush/mode of activation (manual vs. rotating-oscillating, sensitive mode vs. rotating-oscillating, daily clean mode vs. sonic, sensitive mode vs. sonic, power mode) and the brushing force (1 N vs. 2 N) as factors. Separately for enamel and dentin, one-way ANOVAs followed by Scheffe’s (enamel, homogeneous variances) or Tamhane’s (dentin, nonhomogeneous variances) post-hoc tests were performed.

## Results

Enamel and dentin erosive wear (μm, mean ± standard deviation) are presented in Table 1.

**Table 1:** Loss (μm, mean ± standard deviation) of primary bovine enamel and dentin in the different groups including control (erosion only).

Toothpaste	Kind of toothbrush	Brushing force (N)	Enamel loss	Dentin loss
Fluoride	Manual	1	29.2 ± 1.8^b,c^	6.2 ± 3.1^a^
		2	33.2 ± 1.9^b,c,d,e^	5.1 ± 2.3^a^
	Rotating-oscillating, sensitive mode (7,100/min)	1	28.3 ± 2.8^a,b^	5.4 ± 1.6^a^
		2	23.8 ± 1.3^a^	5.7 ± 1.7^a^
	Rotating-oscillating, daily clean mode (7,600/min)	1	34.2 ± 2.6^c,d,e,f^	7.3 ± 2.5^a^
		2	33.3 ± 2.2^b,c,d,e^	7.7 ± 3.6^a^
	Sonic, sensitive mode (16,000/min)	1	31.3 ± 2.6^b,c,d^	6.1 ± 2.3^a^
		2	31.9 ± 1.5^b,c,d^	5.9 ± 2.5^a^
	Sonic, power mode (18,000/min)	1	33.6 ± 2.5^c,d,e^	6.8 ± 3.1^a^
		2	32.3 ± 2.2^b,c,d^	6.5 ± 2.5^a^
Fluoride-free	Manual	1	40.9 ± 2.6^g^	23.9 ± 4.9^b,c^
		2	41.1 ± 2.7^g^	19.6 ± 4.4^b,c^
	Rotating-oscillating, sensitive mode (7,100/min)	1	35.1 ± 2.3^d,e,f^	26.0 ± 5.9^b,c^
		2	38.4 ± 3.5^e,f,g^	28.6 ± 6.6^c^
	Rotating-oscillating, daily clean mode (7,600/min)	1	39.0 ± 1.1^f,g^	17.2 ± 4.4^b^
		2	37.7 ± 1.4^e,f,g^	19.7 ± 4.0^b,c^
	Sonic, sensitive mode (16,000/min)	1	38.9 ± 1.3^f,g^	20.7 ± 4.0^b,c^
		2	35.4 ± 2.3^d,e,f^	25.0 ± 5.3^b,c^
	Sonic, power mode (18,000/min)	1	35.5 ± 2.8^d,e,f^	21.1 ± 4.3^b,c^
		2	42.9 ± 2.2^g^	19.4 ± 6.8^b,c^
Control (erosion only)			38.0 ± 2.3^e,f,g^	18.5 ± 5.0^b,c^

Within enamel and dentin, significant differences between groups are marked with different letters.

Three-way ANOVAs conducted on both enamel and dentin indicated that both the type of toothpaste and toothbrush used were significant factors contributing to substance loss, while brushing force did not show significant effects. The results of the statistical analysis by three-way ANOVAs in enamel and dentin with the p-values of the main effects (toothpaste, toothbrush, brushing force) and their possible interactions are shown in Table 2.

**Table 2:** Results of the statistical analysis by three-way ANOVA in enamel and dentin.

Effect	Enamel p-value	Dentin p-value
Toothpaste	< 0.001	< 0.001
Toothbrush	< 0.001	< 0.001
Force	0.160	0.620
Toothpaste * Toothbrush	< 0.001	< 0.001
Toothpaste * Force	0.005	0.425
Toothbrush * Force	< 0.001	0.023
Toothpaste * Toothbrush * Force	< 0.001	0.142

P-values of the main effects (toothpaste, toothbrush, brushing force) and their possible interactions.

In primary bovine enamel, erosive surface loss in the control group amounted to 38.0 ± 2.3 μm. Brushing with the fluoride toothpaste was able to reduce erosive wear by 15 to 37%, when brushing was performed with the manual toothbrush (1 N), the rotating-oscillating toothbrush sensitive (both 1 and 2 N), the sonic toothbrush sensitive (both 1 and 2 N), and the sonic toothbrush power (2 N). In all other groups brushed with the fluoridated toothpaste, erosive loss was slightly reduced, but was not significantly different from control. Enamel loss in groups that were brushed with the fluoride-free toothpaste was not significantly different from control.

Considering the use of a fluoridated toothpaste and brushing treatment at 1 N brushing force, brushing with the rotating-oscillating toothbrush in sensitive mode caused less wear than brushing with the rotating-oscillating toothbrush in daily clean mode or brushing with the sonic toothbrush in power mode. Within the groups brushed with the fluoridated toothpaste at 2 N brushing force, the rotating-oscillating toothbrush in sensitive mode caused significantly less wear compared to the other groups. Within the same toothbrush and application mode, no significant differences between 1 N and 2 N brushing force were detected except for specimens that were brushed with fluoride-free toothpaste and the sonic toothbrush in power mode.

In primary bovine dentin, erosive surface loss in the control group amounted to 18.5 ± 5.0 μm. Brushing with the fluoridated toothpastes decreased erosive surface loss by 58 to 72% in all groups, while groups that were brushed with the fluoride-free toothpaste were not significantly different from control. Considering the use of a fluoridated or a fluoride-free toothpaste, no significant differences regarding the different toothbrushes (at same brushing force) or the brushing forces (within the same toothbrush) were detected.

## Discussion

This study showed that mainly the kind of toothpaste and also the kind of toothbrush, but not the brushing force (1 N vs. 2 N) affected erosive tooth wear of bovine primary enamel and dentin. Thus, the null hypothesis has to be partially rejected.

The overall design of the study was oriented on a previous study by Chalkidis and coworkers [8] analysing the effect of different children’s toothpastes on erosive wear in primary bovine dental hard tissue. As discussed by Chalkidis and coworkers [8], the experimental setup followed the guidelines for erosion-abrasion experiments [19] with regard to the use of bovine dental hard tissue, erosion, interim storage in artificial saliva, and toothbrushing. With regard to the brushing treatment, three different toothbrushes were chosen (i.e. manual, sonic, and rotating-oscillating) to reflect common toothbrushes for children with different motion action. Specimens were brushed twice daily for 15 s to reflect the frequency and duration of daily toothbrushing by children [17, 24–26], and to reflect the contact time between the toothbrush and one tooth during toothbrushing [19]. As powered toothbrushes are usually applied with less movement compared to manual toothbrushes, the individual brushing frequency (back and forth movement) was adjusted to 20 strokes/min or 80 strokes/min, respectively [21, 22]. Furthermore, two different brushing forces were applied. For manual toothbrushing, both children and caregivers were shown to apply about 2 N brushing force [27, 28], while powered toothbrushes are usually applied at lower brushing force [12]. To allow for better comparison, both manual and power toothbrushes were applied at both forces in this study.

Although the present study followed the recommendations for in-vitro studies on erosion/abrasion [19], the results of the present in-vitro study must be interpreted with caution as in-vitro studies per definition lack external validity and generalisability to clinical situations [29]. Also, potential outcome assessment bias cannot be excluded, as enamel and dentin specimens differ in colour and, thus, can be distinguished from each other.

Confirming the results of previous studies [4, 8], brushing with the fluoridated toothpaste was able to reduce erosive tooth wear in primary enamel and especially in primary dentin, while tooth loss was not different from the controls (erosion only) after brushing with the fluoride-free toothpaste. More recent studies on permanent dental hard tissue have already shown that the preventive effect by the application of fluoridated toothpastes exceeds potential adverse effects (abrasion) from toothbrushing as long as exaggerated brushing is avoided and short brushing treatment simulating clinical conditions is performed [9, 30].

Interestingly, the kind of toothbrush affected erosive tooth wear significantly in enamel and dentin, but overall effects were small and significantly depending on the kind of toothpaste. For permanent teeth, previous studies demonstrated that sonic and rotating-oscillating toothbrushes led to higher wear of eroded enamel and dentin than manual brushes when applied at the same brushing force [21, 22]. Considering that power toothbrushes are usually applied at lower brushing force than manual toothbrushes, brushing with sonic and rotating-oscillating toothbrushes did not increase tooth wear on enamel compared manual brushing. Loss of eroded dentin was even significantly lower when brushed with sonic and rotating-oscillating rather than manual toothbrushes [12]. However, in the present study no clear trend regarding one specific toothbrush or activation mode, respectively, could be seen.

Overall, enamel and dentin losses were not affected by brushing force, independently of the use of powered or manual toothbrushes. Previous studies on the effect of brushing force on eroded permanent enamel [31] and dentin [32] found some significant effects with increased brushing load, but only if brushing force exceeded 2.5 N (enamel) or 2 N (dentin).

## Conclusions

Within the limitations of the present study, erosive tooth wear in the primary dentition might be reduced by using a fluoridated toothpaste. The kind of toothbrush has only a limited and the brushing force has no effect on erosive tooth wear.

## Supporting information

S1 File Dataset. (CSV)
